# Idiopathic basal ganglia calcification associated with cerebral micro-infarcts: a case report

**DOI:** 10.1186/s12883-018-1048-x

**Published:** 2018-04-17

**Authors:** Takuma Nishimoto, Fumiaki Oka, Hideyuki Ishihara, Mizuya Shinoyama, Michiyasu Suzuki

**Affiliations:** Department of Neurosurgery, Yamaguchi University School of Medicine, 1-1-1, Minamikogushi, Ube, Yamaguchi, 755-8505 Japan

**Keywords:** Idiopathic basal ganglia calcification, Cerebral micro-infarcts, Vascular calcification, Single photon emission computed tomography, Cerebrovascular reactivity

## Abstract

**Background:**

Idiopathic basal ganglia calcification (IBGC) is a rare neurodegenerative disorder characterized by symmetric intracranial calcium deposition. We report a patient with IBGC associated with cerebral infarction due to impairment of cerebrovascular reactivity based on single-photon emission computed tomography (SPECT) with acetazolamide challenge.

**Case presentation:**

A 66-year-old male presented with right conjugate deviation, right hemiparesis and total aphasia due to a convulsive seizure. Brain computed tomography showed symmetric calcifications in the bilateral basal ganglia, thalamus, cerebellar dentate nuclei, which were consistent with IBGC. Diffusion-weighted brain magnetic resonance imaging showed multiple small infarctions in the bilateral cerebral subcortical area. In the search for the cause of cerebral infarction, SPECT with acetazolamide challenge revealed heterogeneous impairment of cerebrovascular reactivity in the whole brain, despite the absence of evidence for steno-occlusive changes in proximal arteries.

**Conclusion:**

Cerebrovascular insufficiency due to the lack of elasticity caused by microvascular calcification might have been one of the pathophysiological features of IBGC in this case. Thus, vascular calcification may cause cerebrovascular disturbance and could lead to ischemic stroke in patients with IBGC.

## Background

Idiopathic basal ganglia calcification (IBGC) was first described by Karl Theodor Fahr in 1930 [1] and is also known as Fahr’s disease. IBGC is a rare neurodegenerative disorder characterized by symmetric intracranial calcium deposition mainly in the bilateral basal ganglia and dentate nuclei of the cerebellum in the absence of other causes of secondary calcification. Pathologically, calcium and other mineral deposits are found in the walls of the arterioles, capillaries, small veins and the perivascular space [2, 3]. Patients generally present with chronic and progressive symptoms, such as headache, psychiatric symptoms, epilepsy, dementia and Parkinsonism, and there has been one case report of a patient with IBGC who developed ischemic stroke [4]. Herein, we report a case of IBGC in which multiple small cerebral infarctions developed after general convulsion, and we discuss the possible mechanisms of ischemic stroke in patients with IBGC.

## Case presentation

A 66-year-old male developed general epileptic convulsion and was admitted to our department one hour after onset. His history included epilepsy and hypertension. On admission, blood pressure was 126/84 mmHg and pulse was 82 beats/min with sinus rhythm. Neurological findings included deterioration of consciousness, right conjugate deviation, total aphasia and right hemiparesis with a manual muscle test score of 1/5. The National Institutes of Health Stroke Scale score was 20 points.

**Fig. 1:**
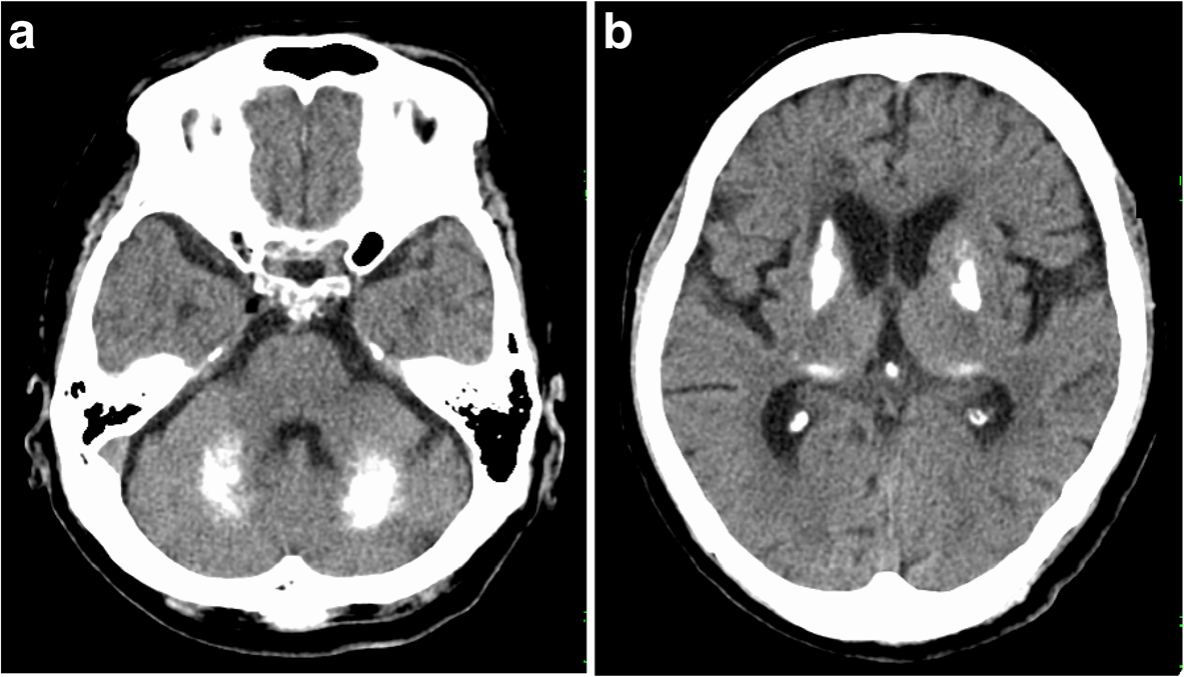
Brain computed tomography (CT). Axial noncontrast CT showing expansive and symmetric high-density lesions involving the cerebellar dentate nuclei (**a**) and bilateral basal ganglia and thalami (**b**)

**Fig. 2:**
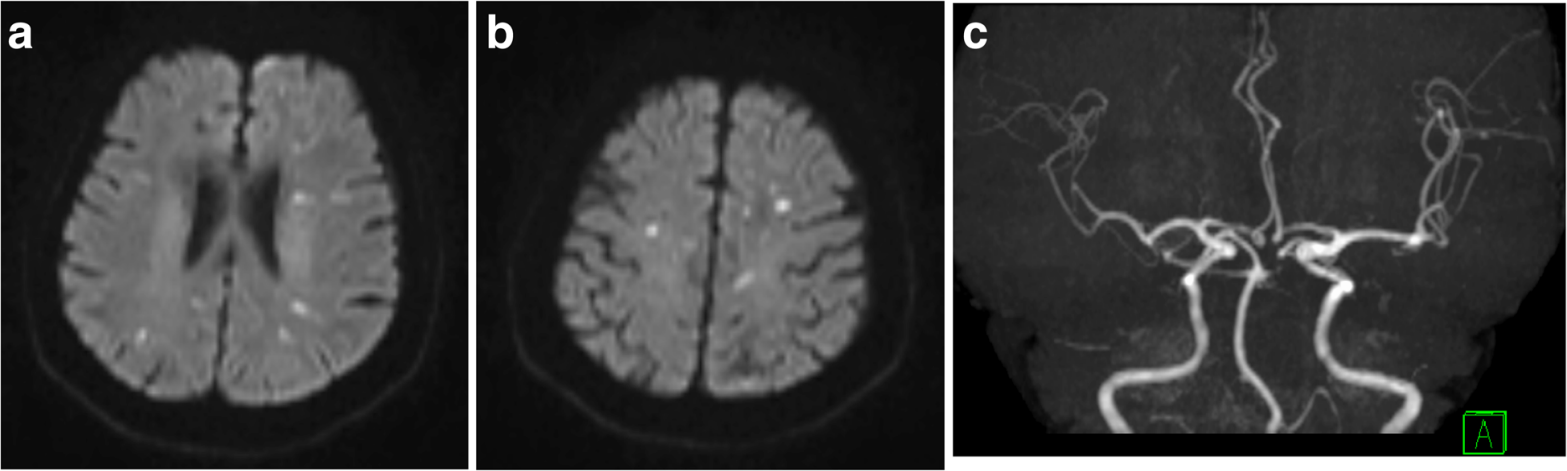
Brain magnetic resonance imaging (MRI). Axial diffusion-weighted brain MRI showing multiple hyperintense spots in the bilateral cerebral subcortical white matter (**a**, **b**). No intracranial arterial occlusion or stenosis was detected by magnetic resonance angiography (**c**)

**Fig. 3:**
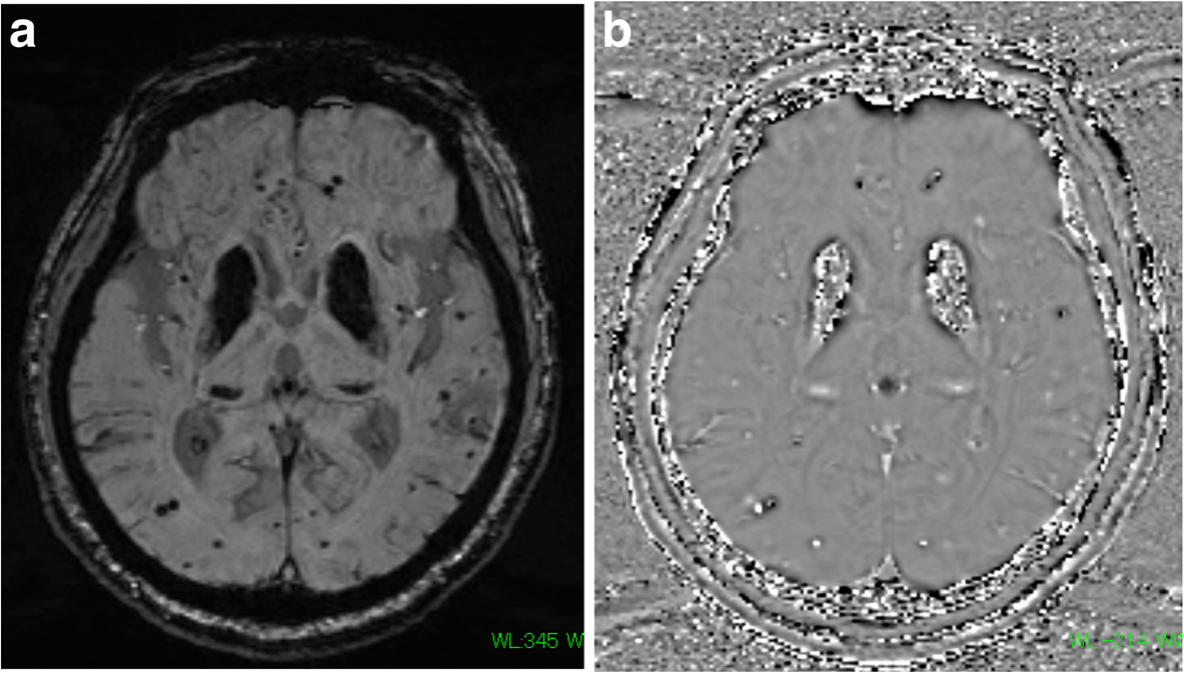
Brain magnetic resonance imaging (MRI). Minimal intensity projection (**a**) and phase images (**b**) on SWI showing multiple hypointense spots in the cerebral sulcus and hypointense lesions coinciding with high-density lesions on CT

Brain computed tomography (CT) showed expansive and symmetric high-density lesions involving the bilateral basal ganglia, thalami and cerebellar dentate nuclei (Fig. 1). Diffusion-weighted brain magnetic resonance imaging (MRI) showed multiple hyperintense spots in the bilateral cerebral subcortical area (Fig. 2a, b), which were indicative of acute infarctions. Magnetic resonance angiography did not show any intra- or extracranial arterial occlusion, stenosis or other vascular abnormality (Fig. 2c). MRI using susceptibility-weighted imaging (SWI) showed hypointense lesions coinciding with high-density lesions in CT imaging and multiple hypointense spots in the cerebral parenchyma (Fig. 3a, b). Hypointense lesions that coincided with the high-density lesions on CT were detected on both minimal intensity projection and phase images on SWI. Moreover, multiple hypointense spots were found in the bilateral cerebral subcortical white matter, which suggested multiple calcifications in the subcortical area.

After admission, we started administration of an anti-epileptic drug and convulsion was stopped. Thereafter, the patient gradually became alert and fully recovered from the neurological deficits within 24 h after admission. One day after admission, electroencephalography showed no apparent epileptic discharge or other abnormal findings. Blood pressure was stable all day at 110 to 130 mmHg (systolic) and 60 to 80 mmHg (diastolic).

Examinations were performed to investigate the cause of intracranial calcification. The serum levels of sodium, potassium, chloride, calcium, albumin, phosphorus, intact PTH, thyroid profile, lipid profile and uric acid were all within normal limits, except for low-density lipoprotein cholesterol (133 mg/dL). Complete cell count, C-reactive protein and erythrocyte sedimentation rate were also normal. Autoimmune tests including antinuclear antibody, anti-single and -double-stranded DNA IgG antibody, rheumatoid factor, cytoplasmic and myeloperoxidase antineutrophil cytoplasmic antibody, and anticardiolipin antibody were all negative or within normal limits. To exclude infection, we examined hepatitis B and C antibodies and human immunodeficiency virus − 1 and − 2 antibodies, and performed a venereal disease test, and all were negative. Laboratory tests and whole body CT showed no evidence of secondary calcification diseases, such as parathyroid disorder, pseudohypoparathyroidism, thyroid disease, other endocrine disease, metabolic disease, collagen diseases, vasculitis, infections (syphilis, hepatitis and HIV) or cysticercosis. Based on these findings, the patient was diagnosed with IBGC.

Concurrently, we performed examinations to investigate the cause of cerebral infarction. Transthoracic echocardiography showed no particular findings. Transesophageal echocardiography revealed a patent foramen ovale; however, there was no evidence of deep vein thrombosis in the lower limbs on contrast CT and ultrasound sonography including Doppler. A 24-h Holter electrocardiogram and monitoring of a continuous 3-lead cable bedside electrocardiogram for 7 days did not show atrial fibrillation or other arrhythmia. Digital subtraction angiography showed no atherosclerotic changes in the proximal arteries.

**Fig. 4:**
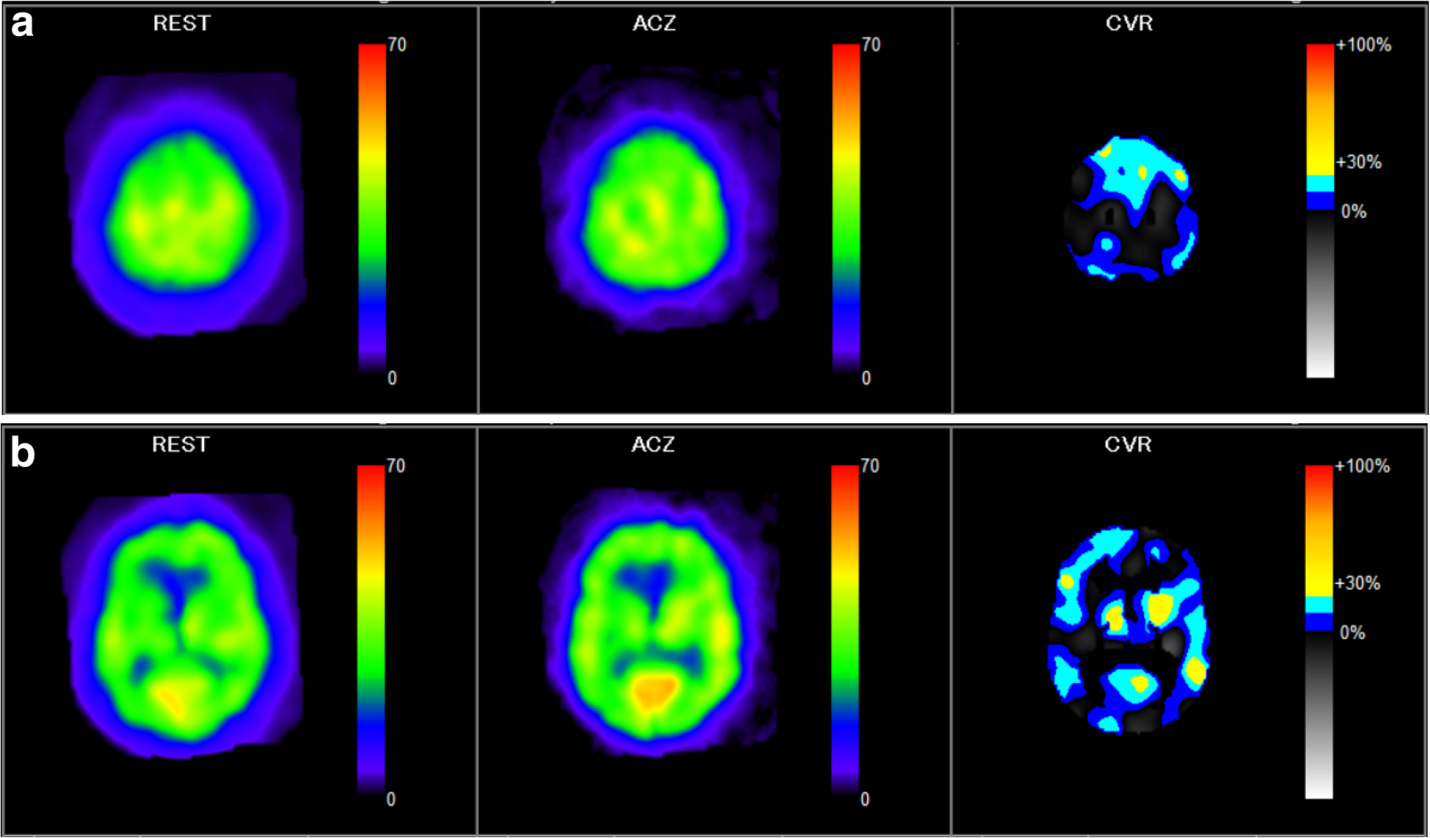
Single-photon emission computed tomography (SPECT). Quantitative CBF assessment with SPECT showed a mild decrease in resting CBF and a severe decrease in CVR over almost all the cerebral area in an acetazolamide-tolerance test. Images are shown at the level of (**a**) the centrum semiovale and (**b**) the basal ganglia. ACZ; acetazolamide, CVR; cerebrovascular reactivity

Quantitative resting cerebral blood flow (CBF) and cerebrovascular reactivity (CVR) were measured by single-photon emission computed tomography (SPECT) in one session using the dual-table autoradiographic method and dual administration of *N*-isopropyl-p-[I-123] iodoamphetamine (^123^I-IMP), as described in detail elsewhere [5]. For SPECT with drug challenge, acetazolamide (17 mg/kg) was administered intravenously. Tomographic images were reconstructed and CBF quantification was performed using the QSPECT image reconstruction package [6]. After image reconstruction, images were processed using NEURO FLEXER. This program automatically measures CBF values at rest and after acetazolamide challenge, and can also calculate CVR, as follows: CVR = 100 × (Diamox CBF- resting CBF) / resting CBF [7]. Surprisingly, resting CBF was decreased slightly, and moreover, CVR was severely impaired heterogeneously at basal ganglia or thalami, which were well-calcified areas, and at all other cortical and subcortical areas of the bilateral cerebral hemispheres, in the absence of steno-occlusive changes in the proximal arteries (Fig. 4).

Thorough blood examinations were also conducted to investigate the possibility of coagulation disorders, such as antiphospholipid antibody syndrome, collagen disease, Protein S and C deficiency, antithrombin III deficiency and tumor markers, but the results were unremarkable. Finally, even though we could not determine the exact cause of cerebral infarction, we concluded that the lack of vascular reactivity might have played a role in causing multiple cerebral infarctions after convulsive seizure in this patient. To prevent secondary attack, the patient was treated with an antiplatelet drug, cilostazol (200 mg/day), and was finally discharged from hospital with no neurological deficits.

## Conclusions

IBGC is a rare neurodegenerative disorder characterized by symmetric intracranial calcium deposition. The common sites of calcification in IBGC are the globus pallidus, putamen, caudate nucleus, internal capsule, dentate nucleus, thalamus, and subcortical white matter [8]. CT and MRI using SWI are useful modalities for detecting calcification in IBGC [9–11]. Sahin et al. reported that SWI phase images, in particular, are comparable to CT for identifying intracranial calcification [12]. In our case, both CT and SWI revealed calcium deposits in the bilateral basal ganglia, thalamus and cerebellar dentate nuclei. Furthermore, SWI showed multiple hypointense spots in the subcortical white matter, which indicated calcium deposition in areas that could not be detected by CT.

Only one case report has shown a clinical relationship between cerebral infarction and IBGC [4]. This report showed young onset of ischemic stroke at the internal capsule, similar to well-known lacunar infarction, in a patient with a IBGC. In contrast, in our case, cerebral infarctions occurred simultaneously in multiple subcortical areas in both hemispheres after a convulsive seizure. Although the mechanism of infarction is not clear in our case, it might be related to impaired CVR throughout the whole brain, as revealed by SPECT. Impaired CVR is a risk factor for stroke [13, 14] and we assume that impaired CVR might have been related to calcification of blood vessels. Vascular calcifications compromise arterial elastance, and calcification in IBGC is mainly observed in the walls of capillary vessels, arterioles, small veins and the perivascular space [2, 3]. Multiple infarctions may have been another cause of impairment of CVR, since a trend for a negative correlation between CVR and the number of lacunar infarctions has been reported [13]. Additionally, since impairment of CVR in hypertensive patients has been reported, history of hypertension in this patient may also be associated with impaired CVR [15]. Based on the MRI and SPECT findings, we postulate that one of the mechanisms of cerebral infarction in our patient with IBGC was a demand-supply mismatch during convulsion. In other words, the convulsive seizure increased the demand for cerebral blood flow, but the demand could not be met due to the decrease in vascular diastolic capacity related to calcification, and this consequently resulted in cerebral infarction.

This is the first reported case of a patient with IBGC with impairment of CVR found on SPECT with acetazolamide challenge. Vascular calcification may cause cerebrovascular disturbance and could lead to ischemic stroke or other phenotypes, such as progressive cognitive impairment, in patients with IBGC. Further studies in a larger numbers of patients are required to understand the pathophysiology of IBGC.

## Abbreviations

^123^I-IMP: *N*-isopropyl-p-[I-123] iodoamphetamine
CBF: Cerebral blood flow
CT: Computed tomography
CVR: Cerebrovascular reactivity
IBGC: Idiopathic basal ganglia calcification
MRI: Magnetic resonance imaging
SPECT: Single-photon emission computed tomography
SWI: Susceptibility-weighted imaging
